# Current Advancements and Limitations of Gene Editing in Orphan Crops

**DOI:** 10.3389/fpls.2021.742932

**Published:** 2021-09-22

**Authors:** Matthew Venezia, Kate M. Creasey Krainer

**Affiliations:** Grow More Foundation, New York, NY, United States

**Keywords:** orphan crops, clustered regularly interspaced short palindromic repeats, gene editing, Cas nuclease, guide RNA

## Abstract

Gene editing provides precise, heritable genome mutagenesis without permanent transgenesis, and has been widely demonstrated and applied *in planta*. In the past decade, clustered regularly interspaced short palindromic repeats (CRISPR)/CRISPR-associated proteins (Cas) has revolutionized the application of gene editing in crops, with mechanistic advances expanding its potential, including prime editing and base editing. To date, CRISPR/Cas has been utilized in over a dozen orphan crops with diverse genetic backgrounds, leading to novel alleles and beneficial phenotypes for breeders, growers, and consumers. In conjunction with the adoption of science-based regulatory practices, there is potential for CRISPR/Cas-mediated gene editing in orphan crop improvement programs to solve a plethora of agricultural problems, especially impacting developing countries. Genome sequencing has progressed, becoming more affordable and applicable to orphan crops. Open-access resources allow for target gene identification and guide RNA (gRNA) design and evaluation, with modular cloning systems and enzyme screening methods providing experimental feasibility. While the genomic and mechanistic limitations are being overcome, crop transformation and regeneration continue to be the bottleneck for gene editing applications. International collaboration between all stakeholders involved in crop improvement is vital to provide equitable access and bridge the scientific gap between the world’s most economically important crops and the most under-researched crops. This review describes the mechanisms and workflow of CRISPR/Cas *in planta* and addresses the challenges, current applications, and future prospects in orphan crops.

## Introduction

Gene editing allows for the precise mutagenesis of a target genome without permanently introducing DNA to the target organism and is directed by site-specific nucleases (SSNs). SSNs, including meganucleases, zinc finger nucleases (ZFNs), transcription activator-like effector nucleases (TALENs), and clustered regularly interspaced short palindromic repeats/CRISPR-associated proteins (CRISPR/Cas), induce targeted double-strand breaks (DSBs) in DNA (Kim et al., 1996; Christian et al., 2010). Targeted DSBs are repaired by endogenous cellular repair mechanisms, non-homologous end joining (NHEJ), and homology directed repair (HDR; Puchta et al., 1996; Puchta, 2004; Jinek et al., 2012). NHEJ is an error-prone pathway in which endogenous repair may result in a short insertion, deletion, or substitution of base pairs (indel) at the site of the DSB. Indels or substitutions can introduce frameshift mutations, alternative stop codons, or codon deletions/insertions, which generally lead to gene knockout (Chakrabarti et al., 2019). With the addition of donor DNA, HDR can result in targeted insertions at the site of the DSB (Zhao et al., 2016; Hahn et al., 2018). Either DNA repair mechanism can occur based on endogenous factors, such as the cell cycle. *In planta*, most repair events occur through NHEJ (Hahn et al., 2018). The use of gene editing with SSNs in plants is well demonstrated, having been applied to model species as well as crops (Townsend et al., 2009; Jiang et al., 2013; Shan et al., 2013; Liang et al., 2014; Odipio et al., 2017). In crops, gene editing has been utilized extensively as both an alternative and companion to conventional breeding (Jia et al., 2017; Lemmon et al., 2018). Early methods of gene editing, before the use of CRISPR/Cas, were expensive and less efficient, relying on protein engineering for their development and improvement (Kim et al., 1996; Christian et al., 2010). As such, only genomes of plants with high scientific (Townsend et al., 2009) and economic importance (Liang et al., 2014) have been edited using these systems. CRISPR/Cas systems have since revolutionized gene editing.

**Figure 1:**
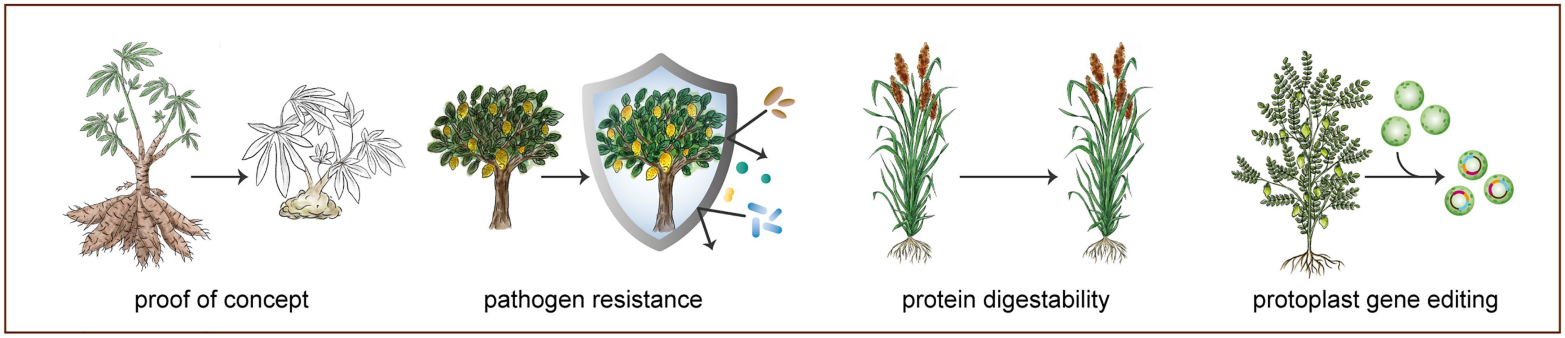
Selection of gene editing applications in orphan crops. Successful demonstration of gene editing in orphan crops ranges from targeting drought response-related genes in chickpea protoplasts to functional knockout of *phytoene desaturase* (*PDS*) in cassava. More agronomically important applications of CRISPR/Cas have produced traits, such as increased protein digestibility in sorghum, achieved through knocking out a highly homologous set of related genes (*k1C* gene family). Another widely studied application of CRISPR/Cas is the functional knockout and promoter editing of *Lateral organ boundaries 1* (*LOB1*) gene in citrus, resulting in citrus canker bacterial resistance. See Supplementary Table 1 for a complete overview of gene editing applications in orphan and underutilized crops.

CRISPR/Cas systems are bacterially derived, RNA guided endonucleases. The Cas endonuclease is guided to the target position on the genome by an engineered guide RNA (gRNA). At this position, a protospacer adjacent motif (PAM) sequence must also be recognized by the Cas enzyme for a DSB to be introduced at the target region (Jinek et al., 2012). The associated cost and labor intensity of designing gRNAs for CRISPR/Cas-mediated gene editing are significantly reduced in comparison with designing protein motifs for ZFNs or TALENs (Bortesi and Fischer, 2015). As a result, CRISPR/Cas systems can be simply, precisely, and cost-effectively applied to any crop, as long as sequence information exists for the target gene (Jiang et al., 2013; Shan et al., 2013; Yin et al., 2017). CRISPR/Cas-mediated gene editing could circumvent the poor public opinion and heavy regulatory process of transgenic approaches and has been estimated to save 9years and USD $10 million in both regulation and crop development when compared to a traditional transgenic crop in the United States (Lassoued et al., 2019). Gene editing with SSNs has now been applied to many agriculturally important, but scientifically and economically ignored crops, or orphan crops (see Figure 1; Supplementary Table 1).

Orphan crops encompass a wide range of staple pulses, cereals, fruits, vegetables, and roots and tubers. There are economically important orphan cash crops, such as cacao, as well as major subsistence crops, such as cassava. Despite local and global agricultural significance, these crops have traditionally been scientifically neglected, underfunded, underutilized, and under-researched since the Green Revolution [Tadele, 2019; African Orphan Crops Consortium (AOCC), 2021]. Although orphan crops are vital to smallholder farmers in less-developed economies, production and yield remain inferior to economically important crops grown in more-developed countries (Tadele, 2014). This is primarily due to the limited number of improved varieties available (Tadele, 2019). Additionally, these crops are vital to smallholders as resilient food sources in a changing climate and represent a large repository of genes for future crop improvement (Mabhaudhi et al., 2019). With food insecurity and population growth disproportionately impacting developing countries, the development of resilient orphan staple crops is imperative. This review specifically focuses on recent advances in CRISPR/Cas-mediated gene editing and applications in orphan crops, highlighting current limitations and the outlook of this powerful technology in the improvement of orphan crops.

## Mechanistic Advances in Gene Editing

### Gene Knockout and Promoter Editing

In gene knockout experiments, a gene product is disrupted, wholly or partially, by targeted mutagenesis with an SSN. If a frameshift mutation or alternative stop codon is introduced as a result of NHEJ, a gene will likely be knocked out and its product disrupted. Currently, most applications of gene editing in orphan crops are based on gene knockouts (Supplementary Table 1). All SSNs (meganucleases, ZFNs, TALENs, and CRISPR/Cas) have been demonstrated to mediate gene knockouts in plants (Puchta et al., 1996; Townsend et al., 2009; Shan et al., 2013; Liang et al., 2014). However, most applications of gene editing currently utilize the CRISPR/Cas system (Xing et al., 2014; Yin et al., 2017; Lemmon et al., 2018; Tian et al., 2018; Liu et al., 2021), and the use of other SSNs in plants, especially orphan crops, is limited in comparison (Supplementary Table 1). Additionally, it is worth noting that previous applications of RNA interference (RNAi), such as the knockdown of genes related to cyanogenic glucoside production in cassava (Jørgensen et al., 2005), may be replicated with gene knockout.

Promoter editing is a precise form of gene knockout, which targets the promoter region of a gene (Peng et al., 2017; Rodríguez-Leal et al., 2017; Liu et al., 2021). In contrast to gene knockout for loss of function, promoter editing serves to modulate gene expression. Targeting single genes, or their promoters, with multiple homologs can result in the upregulation of the redundant genes, as demonstrated in the CLAVATA (CLV)/WUSCHEL (WUS) pathway (Rodriguez-Leal et al., 2019; Liu et al., 2021). Another application of promoter editing is the generation of genetic variation in cultivars by inducing differential gene expression (Rodríguez-Leal et al., 2017). Though not yet widely applied in orphan crops, promoter editing allows for the specific modulation of traits, including for the creation of novel germplasm for breeders, and can aid in developing new paths to domestication for orphan crops (Jia et al., 2016; Rodríguez-Leal et al., 2017; Lemmon et al., 2018). A similar editing technique targets upstream open reading frames (uORFs) allowing for modulation of protein quantity translated at primary ORFs (Zhang et al., 2018).

### Gene Knock-in, Base Editing, and Prime Editing

For more targeted approaches to editing, with fidelity to the individual nucleotide, gene knock-ins, base editing, and prime editing have been developed based on the CRISPR/Cas system. Gene knock-in allows site-specific mutagenesis *via* the HDR pathway of repair following a DSB. Donor DNA of a desired sequence must be introduced concurrently with an SSN to make specific knock-in edits (Puchta et al., 1996; Hahn et al., 2018). However, due to the extremely low frequency of HDR repair in the plant cell, gene knock-in remains inefficient and underutilized (Hahn et al., 2018). Base editing can also be applied to make targeted edits to specific genes. In contrast to knock-ins, specific base pairs can be edited without inducing a DSB in base editing. This is achieved by fusing a catalytically dead Cas9 enzyme (dCas9) or a Cas9 nickase, which retain their ability to specifically bind to DNA *via* a gRNA, to a base editor (Komor et al., 2016). Base editing has been demonstrated and applied *in planta*, including in orphan crops, resulting in herbicide-resistant watermelon (*Citrullus lanatus*; Tian et al., 2018), and mimicking natural polymorphisms for disease resistance in model species (Bastet et al., 2019). Prime editing brings the same benefits of “find and replace” gene editing, though with fewer restrictions through a modified Cas endonuclease and prime editing gRNA (pegRNA) with high specificity (Anzalone et al., 2019). Studies have not yet yielded phenotypic results, though with increasing efforts to improve the efficiency (Lin et al., 2021) prime editing will be widely applied in all crops, including orphan crops.

### Alternate Cas Endonucleases and Multiplexing

The CRISPR/Cas gene editing system was originally developed around a Cas endonuclease derived from *Streptococcus pyogenes* (SpCas9; Jinek et al., 2012). In SpCas9-mediated gene editing, a 19–21bp gRNA is engineered to guide SpCas9 to a target region of DNA, where a PAM sequence (5'-NGG-3') is recognized by the endonuclease. A blunt-cut DSB is then introduced by the endonuclease. The SpCas9-based gene editing system remains the most well studied nearly a decade later and has the largest body of established resources for its utilization as a result (Xing et al., 2014; Stemmer et al., 2015; Liu et al., 2017; Concordet and Haeussler, 2018; Labun et al., 2019). Most applications of gene editing in orphan crops have utilized Cas9 (Supplementary Table 1). Cas9 enzymes derived from other bacteria, for example, *S. aureus*, have increased the efficiency of CRISPR *in planta* (Steinert et al., 2015). However, Cas9 nucleases are limited by the need for a (5'-NGG-3') PAM site (Jinek et al., 2012). To overcome this restriction, Cas nucleases with alternate PAM recognition have been developed. Cas12a, formerly Cpf1 (5'-TTTN-3'), can target T-rich areas of genomes and induce a staggered-cut DSB (Zetsche et al., 2015). A near PAM-less Cas nuclease has also been developed (Walton et al., 2020), allowing gene editing to target regions lacking common PAM sequences. Additionally, some alternate Cas nucleases, namely, the Cas13 family, have the ability to target RNA for interference (Abudayyeh et al., 2016). Cas enzymes have also been modified for alternate applications, such as dCas9 and Cas9 nickases utilized in base editing and gene targeting, which avoid the induction of unwanted DSBs (Komor et al., 2016). Fusions of dCas9 to epigenetic modifiers can also allow epigenetic editing and induce gene activation or repression (Xiao et al., 2019). Another modified endonuclease, Cas9_Trex2, allows more predictable, deletion-only mutations *via* a non-canonical NHEJ pathway (Weiss et al., 2020). To date, of these alternate Cas nucleases, few have been applied in orphan crops: Cas12a has been utilized in *Citrus* spp. (citrus; Jia et al., 2019) and Cas9_Trex2 has been studied in protoplasts and plants of *Setaria viridis* (green foxtail; Weiss et al., 2020).

To target multiple gene homologs or gene families in gene editing, multiplexing must be applied. The only SSN to allow efficient multiplexing is CRISPR/Cas (Xing et al., 2014). Multiplexing with CRISPR/Cas provides simultaneous targeting of multiple genes/alleles by multiple gRNAs (Xing et al., 2014) and has been demonstrated in model plants (Xing et al., 2014; Stuttmann et al., 2021), crops, and orphan crops (Xie et al., 2015; Gomez et al., 2019; Maioli et al., 2020). Multiplexing has further been simplified by utilizing polycistronic gRNA-tRNA for introducing multiple gRNAs. This approach, developed in rice, allows for the introduction of multiple gRNAs processed by cellular tRNA processing (Xie et al., 2015). Alternative approaches for multiplexing include the Csy4 system, which introduces multiple gRNAs interspaced in an array of Csy4 cistrons (Čermák et al., 2017), and the similarly designed ribozyme system, in which gRNAs are flanked by self-cleaving RNA motifs (Gao and Zhao, 2014). Cas12a (Cpf1) also has inherent CRISPR RNA (crRNA) processing capabilities, allowing multiplexing without the need to introduce multiple guides (Zetsche et al., 2017). Future applications of multiplexing in orphan crops can be guided by promising work in model plants (Stuttmann et al., 2021) and the mechanistic advances that have simplified simultaneous introduction of gRNAs.

## Current Methodology of Gene Editing in Orphan Crops

### Gene Target Identification and Guide RNA Design

**Figure 2:**
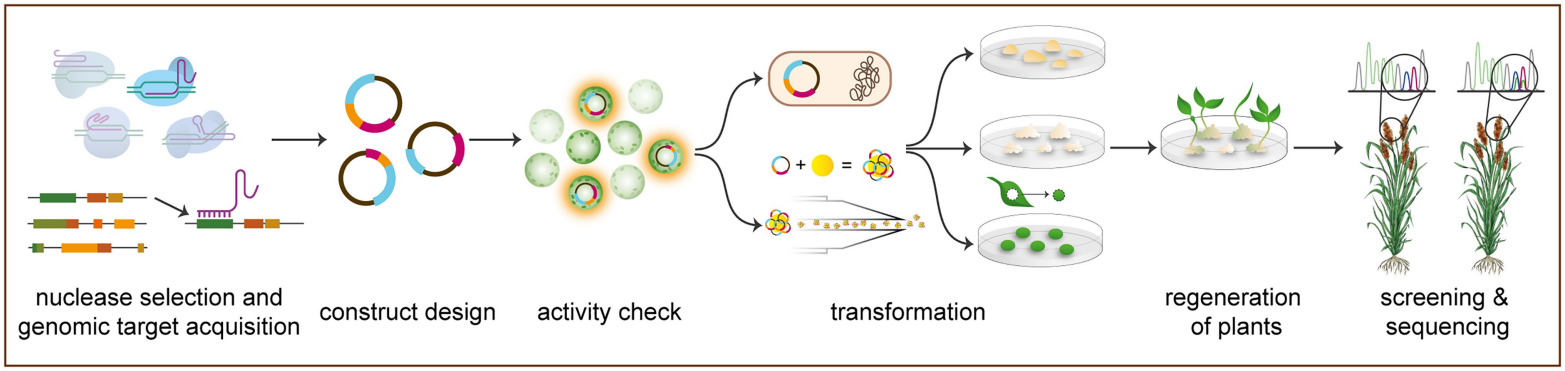
Generalized workflow of CRISPR/Cas-mediated gene editing in orphan crops. The process begins with genomic target acquisition and nuclease selection in which a genomic target is selected based on basic research and a desired gene edited phenotype (see Supplementary Table 1). Gene editing approaches may be based on prime editing, base editing, knock-in, or gene knockout, which are mediated by a variety of nucleases (see “Current Methodology of Gene Editing in Orphan Crops”). Guide RNAs (gRNAs) are subsequently designed for targeting Cas nucleases to site of interest. Next, gRNAs and Cas are cloned under relevant promoters for expression *in planta*, in preparation for bacterial transformation. Gene editing construct activity can be confirmed transiently, usually in protoplasts. Next, the construct is transformed stably into plant material to be regenerated. Transformed plants are subsequently regenerated from callus and selected *via* antibiotic or herbicide resistance. These regenerated primary transformants are then screened and sequenced to determine successful target gene editing. All transgenic DNA can finally be segregated out through breeding to achieve transgene-free gene edited plants (not pictured).

Designing gRNAs for a CRISPR/Cas gene editing application in any crop involves two major steps: acquiring a genomic target and designing a complementary guide sequence (see Figure 2 for an overview of the methodology outlined in this section). To acquire a genomic target, sequence information of the target gene must be available, and a whole genome is desirable to assess off-target activity. Genome browsers, such as NCBI (https://www.ncbi.nlm.nih.gov), Phytozome (https://phytozome.jgi.doe.gov/pz/portal.html), and Ensembl Plants (https://plants.ensembl.org/index.html), provide interactive access to available sequenced genomes, including many orphan staple crops, such as *Manihot esculenta* (cassava), *Sorghum bicolor* (sorghum), and *Vigna unguiculata* (cowpea). The African Orphan Crops Consortium (AOCC) has the ambitious aim to sequence 101 orphan crop genomes [Hendre et al., 2019; African Orphan Crops Consortium (AOCC), 2021] and has published eight new orphan crop genome assemblies to date (Hendre et al., 2019), with four more expected shortly [African Orphan Crops Consortium (AOCC), 2021]. Additionally, many orphan crops have been sequenced by independent research efforts and are not yet available on traditional genome browsers or the AOCC, including *Cajanus cajan* (pigeonpea; Varshney et al., 2012) and *Digitaria exilis* (white fonio; Wang et al., 2021). As genome sequencing methods have improved and become less expensive, more sequenced and annotated orphan crop genomes have become publicly available. Thus, molecular work, including gene editing, in these traditionally neglected species has become more facile. Gene target identification, however, still remains a minor bottleneck for all crops. Potential genes of interest can be determined through gene ontology in related species, previous studies, or widely established pathways. Thus, the reason for continued mechanistic studies in model species to provide understanding of potentially conserved gene function, pathways, and families.

The specific location of the target region within a given genome depends on the method employed and the desired phenotype. For functional gene knockout, the gRNA target sequence should be located within an exonic, translated region, within the first half of the gene (Kaur et al., 2018). UTRs, introns, exon-intron junctions, intergenic regions, and exons furthest from the start codon should be avoided. For modulation of gene expression, gRNAs may target the promoter region or upstream ORF of a target gene. In base editing, gRNAs target the region containing the nucleotide(s) to be exchanged by a cytidine deaminase or similar nucleotide-swapping protein (Rees and Liu, 2018). In prime editing, the gRNA target region is also specific to the nucleotide(s) targeted for exchange; however, the prime editing complex involves a prime editor protein as well as a reverse transcriptase protein. To note, prime editing gRNAs (pegRNAs) are significantly longer than gRNAs utilized for gene knockout or base editing (Anzalone et al., 2019). There is a plethora of available resources to aid in gRNA design, for example, CRISPOR (Concordet and Haeussler, 2018), CCTop (Stemmer et al., 2015), CHOPCHOP (Labun et al., 2019), and CRISPR-P (Liu et al., 2017). These resources allow for the selection of a target genome, the desired Cas nuclease and associated PAM sequence, and target gene sequence input. gRNAs for knockout, knock-in, or base editing can be suggested, and these tools also screen for individual gRNA efficiency and potential off targets. Similar resources are also available for prime editing, for example, PlantPegDesigner (Lin et al., 2021). gRNA design and off-target minimization have been extensively reviewed (Hahn and Nekrasov, 2019; Manghwar et al., 2020). To ensure efficient first steps in future orphan crop gene editing experiments, a focus on the development of genomic and transcriptomic resources for orphan crops, as well as on communication and collaboration to ensure existing gRNA design resources include orphan crop genomes, is necessary.

### Construct Design and Transient Confirmation

Following gene identification and gRNA design, cloning and transformation of plasmid constructs(s) containing the components to express Cas nucleases and gRNAs are necessary for gene editing *in planta* (Figure 2). The Cas nuclease cloned is dependent on the nature on the desired edits (see “Mechanistic Advances in Gene Editing”: Modified and Alternate Cas Endonucleases). Promoters for expressing Cas9 and gRNAs should be based on crop to be transformed and codon optimized accordingly. RNA polymerase (Pol) III promoters, U3 and U6, are utilized for gRNA expression, whereas Cas9 is expressed under Pol II, CaMV35S, or species-specific Ubiquitin (Hahn et al., 2020). Online resources are available for aiding construct design, including SnapGene (www.snapgene.com) and Benchling (www.benchling.com). Modular cloning systems have also greatly simplified the assembly of large multigene constructs for gene editing in crops, making CRISPR more accessible in orphan crops (Čermák et al., 2017; Hahn et al., 2020).

Experimental validation of these constructs is possible, through optional, by transient expression or protoplast transformation (Figure 2) and ensures construct activity prior to more labor-intensive, stable transformation. Transient systems have been utilized in species amenable to transient transformation to generate edits *in planta* without stable transgenesis (Chen et al., 2018). Few plant species are amenable to transient transformation in shoot tissue, however, and the use of this technique is primarily limited to the *Solanaceae* as a result (Chen et al., 2018). An alternate route to *in planta* transient confirmation is a detached leaf assay. Traditionally utilized to study plant pathogens, these systems allow confirmation and experimentation without utilizing a whole *planta* approach. Detached leaf assays for transient gene editing are relatively novel and have been developed for cacao (Fister et al., 2018) and cowpea (Juranić et al., 2020). Protoplast transient systems have also proven suitable for rapidly confirming gene editing construct activity (Lin et al., 2018). After transformation or protoplasts with the experimental construct, high-throughput screening methods, such as restriction fragment length polymorphism (RFLP), can be applied to these systems (Odipio et al., 2017; Lin et al., 2018; Huang et al., 2020; Wu et al., 2020; Badhan et al., 2021). Attempts at protoplast isolation in some crops, such as cowpea, have been unsuccessful (Juranić et al., 2020). It is important to note that the transient confirmation process is advantageous but wholly optional. Most guides produced through the previously mentioned online software are functional, and the academic standard of at least two gRNAs per gene eliminates most concern of low editing efficiency (Liu et al., 2017; Concordet and Haeussler, 2018).

### Transformation, Regeneration, and Screening

**Figure 3:**
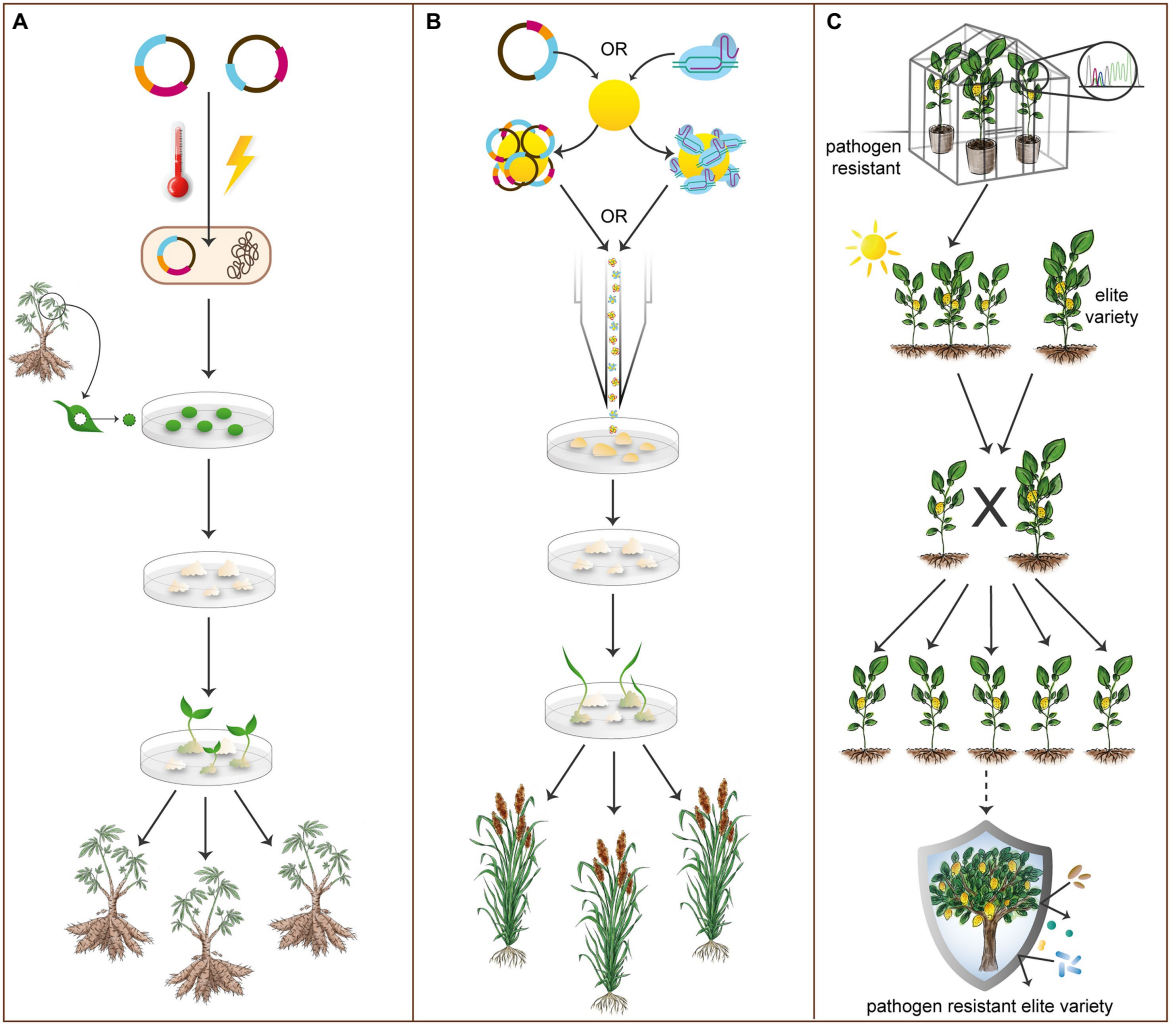
Transformation, regeneration, and breeding methods in orphan crops. **(A)** An overview of *Agrobacterium*-mediated transformation. Constructs are first transformed into agrobacteria. Transformed agrobacteria are then co-cultivated with callus tissue from plant material derived from the target organism. Various types of plant material can be utilized, including leaf explants (shown here). Following transformation and callus induction, shoot and root tissue is then regenerated. As shown, most root/tuber crops are predominantly transformed by *Agrobacterium*-mediated methods (see Supplementary Table 1). **(B)** An overview of biolistic bombardment. Constructs or RNPs are loaded into gold particles and a biolistic particle-based delivery system is utilized to bombard the plant material. Plant material is then regenerated after callus tissue induction. This method of transformation is particularly employed for species considered recalcitrant to *Agrobacterium*-mediated methods. As shown, cereal crops, such as sorghum, may be transformed by biolistic bombardment, though this method is less prevalent than *Agrobacterium*-mediated transformation in orphan crops (see Supplementary Table 1). **(C)** A simplified representation of breeding methods utilized to allow the introgression of gene edited alleles from lab varieties into grower-preferred varieties. Transgene-free gene edited plants that exhibit a desired trait (disease resistance, shown here) are crossed with elite, grower-preferred varieties in marker-assisted breeding to produce an elite variety with the desired gene edited allele. In this manner, gene edited alleles may be introgressed into elite lines considered recalcitrant to conventional transformation methods.

In contrast to transient transformation, stable transformation allows for permanent gene edits, resulting in novel, heritable alleles (Altpeter et al., 2016). The most commonly adopted stable transformation methods in plants include *Agrobacterium*-mediated transformation and biolistic bombardment (Figures 3A,B). Most stable transformation protocols include steps for collecting and wounding plant tissues, agroinfecting or biolistically bombarding these tissues, and then inducing callus growth and eventual differentiation and regeneration into shoot and root tissue (Niklaus et al., 2011; Altpeter et al., 2016; Do et al., 2018). Multiple types of plant tissues can be utilized for stable transformation, all with the goal of inducing callus tissue growth (Supplementary Table 1). Each crop is transformed uniquely, but within clades, methodologies overlap. Many current root/tuber transformation protocols (see Figure 3A) utilize some type of explant material and an *Agrobacterium*-mediated methodology (Niklaus et al., 2011; Wang et al., 2019). Most cereal transformation protocols (see Figure 3B) utilize immature embryo as transformable material and may rely on either biolistic bombardment (Liu et al., 2019a) or *Agrobacterium* (Do et al., 2018) for plasmid delivery. Notably, CRISPR/Cas can be delivered as ribonucleoprotein (RNP) by biolistic bombardment, which has been demonstrated in important crops, including *Triticum aestivum* (wheat; Zhang et al., 2016), *Zea mays* (maize; Svitashev et al., 2016), and *Lactuca sativa* (lettuce; Park et al., 2019). This process avoids bacterial cloning, as well as crossing or segregation to remove construct transgenes from edited plants but comes at a significant financial cost.

Although orphan crops are chronically underfunded, many transformation protocols are available. Both biolistic bombardment and *Agrobacterium*-mediated transformation have been demonstrated in sorghum, the most well-studied orphan cereal (Che et al., 2018; Do et al., 2018; Liu et al., 2019a; Figure 3B). Cassava is the most transformed orphan tuber crop, having been efficiently transformed and regenerated through *Agrobacterium*-mediated methods (Niklaus et al., 2011). Importantly, genotype non-specific methods of transformation have also been developed for the crop (Lentz et al., 2018). Cowpea is the only orphan pulse crop to be transformed efficiently; *via Agrobacterium*-mediated methods, efficient transformation and regeneration rates were achieved (Bett et al., 2019; Che et al., 2021). Many orphan fruit crops, such as citrus, also have viable methods for transformation and regeneration (Donmez et al., 2013; Debernardi et al., 2020).

Throughout the regeneration process, likely transgenic plants are continually screened for by exposing regenerants to a selective antibiotic or herbicide, a resistance gene for which is included in the gene editing construct. Gene edits can be confirmed in primary transformants by RFLP, if the gene edit disrupts the enzyme restriction digestion site (Lin et al., 2018), T7e1 endonuclease, which detects mismatches in DNA strands during PCR (Li et al., 2018a), or barcode sequencing (Smith et al., 2010). Next generation or Sanger sequencing can determine the exact nature of gene edits and should be utilized for validation (Lin et al., 2018; Zsögön et al., 2018; Liu et al., 2021). To note, gene edited crops must go through multiple generations of outcrossing to ensure transgenic DNA is no longer present to be considered by regulatory bodies as a molecular breeding method.

## Applications of Gene Editing in Orphan Crops

### Trial Applications and Phenotypic Controls

Conventional applications of gene editing in orphan crops follow a workflow of genetic target acquisition, construct design and cloning, transformation, regeneration, and screening (See “Current Methodology of Gene Editing in Orphan Crops”; Figure 2). Ideally, the first application of gene editing to a particular species outlines a successful iteration of this workflow, including a transformation and regeneration protocol, a baseline efficiency of transformation, regeneration, and editing and targets a particular gene or multiple genes for editing, often a phenotypic control. The most notable phenotypic control is a knockout of a gene coding for phytoene desaturase (PDS) or a similar enzyme. *PDS* knockout has been applied in multiple orphan crops, including citrus (Jia and Wang, 2014; Zhang et al., 2017; Dutt et al., 2020; Huang et al., 2020), cassava (Odipio et al., 2017), *Dioscorea* spp. (yam; Syombua et al., 2021), watermelon (Tian et al., 2017), *Musa* spp. (banana/plantain; Kaur et al., 2018), *Actinidia chinensis* (kiwifruit; Wang et al., 2018), *Cucumis melo* (melon; Hooghvorst et al., 2019), *Fortunella hindsii* (Hongkong kumquat; Zhu et al., 2019), and sorghum (Liu et al., 2019a). Additionally, *PDS* knockout has been achieved in the first application of CRISPR in *Setaria italica* (foxtail millet) protoplasts (Lin et al., 2018). When PDS is knocked out, a photobleached/leaf-whitening phenotype is observed in edited regenerated plants due to the interruption of the highly conserved carotenoid biosynthetic pathway (Odipio et al., 2017; Tian et al., 2017). Photobleached plants do not survive to maturity and as a result, this phenotype is not heritable.

Other phenotypic controls involve heritable traits, such as flowering time, plant height, and root morphology. In sorghum, flowering time was modulated by CRISPR/Cas-mediated knockout of two related genes, *FLOWERING TIME* (*FT*) and *Gibberellin 2-oxidase 5* (*Ga2ox5*), resulting in heritable targeted mutagenesis (Char et al., 2020). In green foxtail, a wild relative of foxtail millet, two pairs of highly linked genes involved with male sterility were targeted by multiplex editing. More reliable deletion-only mutations for knockout were also achieved with the Cas9_Trex2 system in this trial application (Weiss et al., 2020). In cowpea, the *Symbiosis receptor-like kinase* (*SYMRK*) gene was knocked out to produce a heritable phenotypic effect on root morphology and mycorrhizal symbiosis (Ji et al., 2019). The 9*-cis-EPOXYCAROTENOID DIOXYGENASE4* (*NCED4*) gene in lettuce was knocked out, allowing for germination at high inhibitory temperatures and producing a selectable edited phenotype (Bertier et al., 2018). Some trial applications have also utilized protoplast systems, including the first application of CRISPR/Cas to chickpea, in which two genes associated with drought tolerance, *4-coumerate ligase* (*4CL*) and *Reveille 7* (*RVE7*), were successfully edited (Badhan et al., 2021). Other trial applications bypass phenotypic controls and protoplast systems entirely, modulating important traits based on basic research in model organisms or well-studied crops (Chandrasekaran et al., 2016; Varkonyi-Gasic et al., 2019; Wang et al., 2019; Maioli et al., 2020). Regardless of the trait or phenotypic control, trial applications are vital to orphan crop gene editing as they establish crop-specific workflows and protocols, streamlining future, and trait-centered applications.

### Disease Resistance

CRISPR/Cas has successfully been applied to modulate disease resistance in crops, primarily by targeting susceptibility genes (S genes) for knockout. One notable S gene is *Lateral organ boundaries 1* (*LOB1*) in *Citrus*. Knocking out, as well as promoter editing, of *LOB1* with CRISPR/Cas has led to increased resistance to citrus canker, a major bacterial infection affecting the many species, hybrids, subspecies, and cultivars of *Citrus* (Jia et al., 2016, 2017, 2019; Peng et al., 2017). Similarly, knockout of the *psk1* gene in *C. lanatus* conferred resistance to *Fusarium oxysporum* f.sp. *niveum*, a prominent fungal pathogen of the watermelon to which few cultivated varieties are resistant Zhang et al. (2020). In cacao, an analogous result was achieved by knocking out the *NPR3* gene for increased resistance to fungal pathogens (Fister et al., 2018). Another notable and well-conserved crop S gene is a gene encoding a eukaryotic initiation factor (eIF), specifically in the 4E and 4G family. When an *eIF4* gene or homolog is knocked out in plants, a phenotype of broad-spectrum potyvirus resistance is observed (Rodríguez-Hernández et al., 2012; Chandrasekaran et al., 2016; Gomez et al., 2019). Economically important plant pathogens impacting orphan crops, such as cassava brown streak disease in cassava (Gomez et al., 2019) and *Zucchini yellow mosaic virus* in cucumber (Chandrasekaran et al., 2016), caused fewer symptoms in plant lines where an *eIF4* gene or homolog was knocked out. Given the well-conserved nature of the *eIF4* gene, and previous applications of RNAi to this S gene in other crops, namely, melon (Rodríguez-Hernández et al., 2012), many additional orphan crops could be edited for broad- spectrum potyvirus resistance in this manner. These susceptibility genes were originally characterized by basic research (Robaglia and Caranta, 2006) and more susceptibility genes may be identified through further basic research in model species for future application in orphan crops. In addition, mimicking natural polymorphisms in S genes with base editing has been shown to lead to resistance without entirely knocking out the gene in model plant species, a promising result for future applications of gene editing for disease resistance in orphan crops (Bastet et al., 2019).

### Nutrition

Nutrition-related traits have been modulated with CRISPR/Cas-mediated gene editing in orphan crops by targeting genes in pathways controlling products that limit potential nutrient availability. In *Ipomoea batatas* (sweet potato), *Granule-bound starch synthase I* (*GBSSI*) and *Starch branching enzyme II* (*SBEII*) were knocked out using CRISPR/Cas to produce a plant with decreased complex starch biosynthesis, increasing the nutritional availability of more digestible sugars (Wang et al., 2019). Related genes in cassava, *Protein targeting to starch 1* (*PTST1*) and *GBSS*, were also targeted for knockout by CRISPR/Cas, which resulted in a similar phenotype (Bull et al., 2018). In sorghum, downregulation of Alpha-Kafirins by CRISPR/Cas-mediated knockout of the Alpha-Kafirin gene family (*k1C*) resulted in increased protein digestibility (Li et al., 2018a). Notably, in this study, over 20 genes were targeted by a single gRNA due to the high homology among the genes in this family. Editing of uORFs associated with the *GDP-L-galactose phosphorylase 1* (*GGP1*); *GDP-L-galactose phosphorylase 2* (*GGP2*) genes in lettuce led to a significant increase in the vitamin C content of mutants and additionally improved oxidative stress tolerance (Zhang et al., 2018). As part of a broader effort aimed at *de novo* domestication of *Solanum pimpinellifolium* (wild tomato), knock out of *LYCOPENE BETA CYCLASE* (*CycB*) yielded plants with substantially increased lycopene content without affecting beta carotene levels (Zsögön et al., 2018). Beta carotene levels in banana were similarly increased through CRISPR/Cas-mediated knockout of the *LYCOPENE EPSILON CYCLASE* (*LCYε*) gene (Kaur et al., 2020). Classic applications of RNAi to orphan crops for nutrition traits also outline potential applications of gene editing in orphan crops. For example, in cassava, RNAi was utilized to diminish the endogenous production of cyanogenic glucosides (Jørgensen et al., 2005). CRISPR/Cas-mediated knockout of the same gene products targeted by RNAi will similarly lead to a decrease in the toxin content of cassava tubers.

### Domestication and Breeding

Domestication-related traits are becoming an exciting application of CRISPR/Cas in plants, which could provide advantageous cultivars more rapidly than through conventional breeding practices. In *Physalis pruinosa* (ground cherry), an orphan crop and member of the *Solanaceae*, domestication-related traits were improved with gene editing (Lemmon et al., 2018). Genes related to yield and shoot architecture were targeted, leading to an increased yield and more compact growth habit (Lemmon et al., 2018). Multiple domestication-related traits have also been targeted by gene editing in African rice landraces (Lacchini et al., 2020) and wild tomato (Zsögön et al., 2018), with success in improving yield quantity and quality. Additionally, a path to domesticate wild allotetraploid rice (*Oryza alta*) has been recently elucidated by CRISPR/Cas-mediated gene editing (Yu et al., 2021). Improvement of domestication traits allows for the development of new varieties by breeding and the preservation of important wild traits, such as disease resistance, in novel and newly domesticated orphan crop cultivars. This is especially pertinent in orphan crops, as many have undesirable traits, such as lodging and low yields in most tef cultivars (Tadele, 2019). *De novo* domestication approaches have indicated that improving plant architecture and yield is possible through gene editing, enabling the improvement of agronomically important traits in these neglected crops.

Other traits, especially those important for consumers and growers, have been modulated in orphan crops. In eggplant, multiple related genes in the *Polyphenol oxidase* (*PPO*) family were simultaneously targeted for editing, leading to a decreased-browning phenotype in fruit (Maioli et al., 2020). This application has been similarly achieved in other members of the *Solanaceae*, namely, the tomato, and may also be achieved in newly sequenced crops of the same clade, such as the African eggplant (Song et al., 2019). In watermelon, targeted base editing of the *Acetolactate synthase* (*ALS*) gene resulted in herbicide-resistant plants, an important trait for growers and breeders (Tian et al., 2018). This remains one of the few applications of base editing in orphan crops. Improvement of traits benefitting the consumer and grower demonstrates progress in developing more economically viable cultivars of orphan crops through gene editing, an area in which orphan crops severely lag behind economically important crops.

Other studies have improved single domestication- and breeding-related traits in already domesticated varieties to overcome obstacles related to plant growth habit and breeding. Notably, more compact growth habits were achieved with CRISPR/Cas-mediated gene editing in banana by editing the *Gibberellin 20-oxidase 2* (*GA20ox2*) gene (Shao et al., 2020). A similar result was demonstrated in kiwifruit, by editing the *CENTRORADIALIS* (*CEN*) and *CENTRORADIALIS 4* (*CEN4*; Varkonyi-Gasic et al., 2019). Editing of the *CEN* and *CEN4* genes in kiwifruit also resulted in plants with a decreased flowering time, a beneficial trait for growers and breeders. Gene editing of endogenous banana streak virus (eBSV) present in the B genome of banana/plantain was shown to inactivate eBSV and diminish symptoms of endogenous infection under drought conditions, overcoming a prominent breeding problem in this orphan crop (Tripathi et al., 2019). In cucumber, gynoecious plants for more efficient breeding were created through gene editing of the *WIP1* gene (Hu et al., 2017). Another application to aid in breeding, as well as basic research, is the creation of a haploid inducer line *via* a functional knockout of the *MATRILINEAL* (*MTL*) gene in foxtail millet (Cheng et al., 2021). Improvement of these traits shows the potential of gene editing to overcome obstacles in orphan crop breeding and its ability to directly introduce precise modifications to alleles in elite lines.

In addition to improving traits and providing a path to domestication, editing domestication- and breeding-related traits also provides novel germplasm to breeders for further improvement of existing varieties of orphan crops. CRISPR/Cas can also create allelic diversity by targeting trait-specific loci, providing novel alleles for breeders in elite germplasm (Rodríguez-Leal et al., 2017; Lemmon et al., 2018). The interplay between molecular and conventional breeding has expanded in scope with marker-assisted breeding (Figure 3C). This technique allows for the introgression of favorable gene edited alleles from lab or wild strains into grower-preferred or elite germplasm, which are sometimes recalcitrant to traditional methods of transformation (Dempewolf et al., 2017; Cobb et al., 2019). The timeline for breeding in this manner can be accelerated by including an exogenous flowering time locus within the gene editing construct, to be segregated out during the breeding process, as has been demonstrated in cassava (Bull et al., 2018). Further advances in orphan crop breeding programs, such as speed breeding (Chiurugwi et al., 2019), additionally accelerate the process of crop improvement and breeding.

## Limitations of Gene Editing in Orphan Crops

### Mechanistic Constraints of CRISPR/Cas

The application of CRISPR/Cas has inherent limitations in any crop. Firstly, if a genome sequence is unavailable or unassembled it is impossible to identify potential targets of interest for editing or assess off-target activity of gRNAs (Hahn and Nekrasov, 2019). Without a sequence for the gene of interest, it is also impossible to design the complementary gRNA sequences necessary for directing Cas nucleases to the target site. Off-target activity of CRISPR/Cas is also a primary concern. Off-target activity has been observed *in planta* with as many as six base mismatches between gRNA and off-target loci, though it is most common with three or fewer base mismatches (Modrzejewski et al., 2020). However, it is the location of the mismatches that is most relevant: If base mismatches fall outside of the 8–12bp adjacent to the PAM sequence, off-target activity becomes more common (Modrzejewski et al., 2020). To overcome off-targeting concerns, online gRNA design resources can be utilized to select candidates with minimal off targets (Concordet and Haeussler, 2018; Hahn and Nekrasov, 2019). These resources can also be utilized to avoid candidate gRNAs with low efficiency or incompatibility under Pol III, and with low GC content (Concordet and Haeussler, 2018).

The need for a PAM sequence restricts editing certain areas of any genome and limits the number of potential gRNA candidates. This particularly restricts CRISPR/Cas base editing, for which it is necessary to target nucleotides within a base editor catalytic window (usually 4–5bp, but up to 17bp) within the vicinity of a PAM sequence, meaning targeting highly specific base edits to many areas of a genome is currently impossible (Mishra et al., 2020). A near PAM-less Cas enzyme has recently been developed (Walton et al., 2020) and was applied in plants (Ren et al., 2021) to help overcome this limitation. However, this alternate Cas enzyme has not yet been applied in orphan crops nor has it been applied with base editors in plants. Results in rice protoplasts also indicate far lower editing frequencies when compared to Cas9 (Ren et al., 2021). Cas proteins with alternate PAM sequences can be utilized to extend the scope of possible target sites without sacrificing editing efficiency, such as Cas12a for T-rich genomic regions (Zetsche et al., 2015). While this solution can expand the number of targets for knockout and base editing approaches, these alternate endonucleases have not been studied to the same extent as SpCas9, and base editors have not been tested at all with many Cas variants.

A further limitation in base editing is the restriction of specific nucleotide changes (Mishra et al., 2020). Prime editing could overcome these limitations by allowing highly targetable “find and replace edits,” which can facilitate a swap from any one base to any other base; however, initial findings suggest low editing efficiencies, especially when attempting generation of stable transformants (Lin et al., 2020). As neither base editing nor prime editing has been applied widely in orphan crops to date, these limitations should guide their future application. Targeted insertions *via* the HDR pathway could also overcome these limitations in base editing; however, HDR is observed at extremely low frequency, thus limiting the capacity for precise knock-in gene edits *via* donor DNA (Hahn et al., 2018). Large sample sizes will be necessary to address the low occurrence of HDR, impacting the associated labor and costs. To date, only model plant species (Hahn et al., 2018) and economically important crop species (Li et al., 2018b) have undergone knock-in gene editing as a result. It is also important to note that crops derived from knock-in editing are considered transgenic and subsequently must comply with local and international regulatory policies associated with transgenics.

### The Transformation and Regeneration Bottleneck

Molecular and genetic advances in all plants are restricted by the bottlenecks of transformation and regeneration. Neither major method for stable transformation (*Agrobacterium*-mediated or biolistic bombardment) is applicable to all crops. Though significant discoveries and improvements have been made over the past three decades, there are still issues to be overcome, including (1) there are few genotypes within a given crop species in which *Agrobacterium*-mediated transformation is efficient; (2) low precision, high probability of off-target genome damage and tissue damage in biolistic bombardment (Liu et al., 2019b); (3) low rate of stable transformation events occurs with both methodologies; (4) difficulties in developing efficient *Agrobacterium*-mediated transformation and regeneration protocols in monocot crops (cereals, such as maize and sorghum, are notoriously difficult to transform, with complications arising in both methodologies for each of these crops); and (5) long tissue culture periods required for regeneration of plants from transformed tissue (Altpeter et al., 2016). A novel strategy avoids many of these limitations by utilizing carbon nanotubes to transform plant cells. This method shows promise for the generation of stable, gene edited lines, though more basic research is needed before it can be widely applied (Demirer et al., 2019).

Crop-specific improvements have been developed, for example, the use of surfactant in sorghum transformation (Che et al., 2018) or sonication in legume transformation (Bett et al., 2019). Additionally, overexpression of morphogenic regulators, *BABY BOOM* (*BBM*) and *WUS*, during transformation increases callus tissue proliferation and subsequent regeneration efficiency in maize (Lowe et al., 2016). Similar results have been achieved by expressing GROWTH-REGULATING FACTOR/GRF INTERACTING FACTOR in wheat and citrus (Debernardi et al., 2020). These improvements, however, have not dramatically increased transformation efficiency.

For orphan crops, transformation and regeneration present a particularly challenging obstacle, as there has been little research on the optimization of these methods in many underfunded crops. Despite the successes outlined in Current Methodology of Gene Editing in Orphan Crops, most other orphan crops are not as well studied as those previously mentioned, and transformation for most orphan crops remains inefficient or unexplored. Additionally, transformation protocols for difficult to transform crops are largely genome-specific (Do et al., 2018; Che et al., 2021), limiting the capacity of genome editing in grower-preferred varieties. As such, the development of efficient transformation protocols that can be applied to diverse genomes within a species of orphan crop, and improvement on current transformation protocols to increase efficiency, are necessary steps needed for the improvement of orphan crops with gene editing.

### Complex Traits and Upregulation

The majority of orphan crop genome editing studies have resulted in the loss-of-function of target gene products (Supplementary Table 1). Some gene knockouts have unintended phenotypic consequences because of the multifunctionality of gene products. For example, a decrease in yield has been observed when a gene (*IPK1*) coding for the enzyme (Inositol 1,3,4,5,6-pentakisphosphate 2-kinase) involved in maize phytic acid biosynthesis is knocked out (Liang et al., 2014; Singh et al., 2020). A more effective strategy for *IPK1*, and its orthologs in related orphan crops, may be base editing or prime editing which can disrupt the active site of the enzyme without altering other protein domains (Lin et al., 2020; Singh et al., 2020). However, these more targeted strategies have yet to be widely applied in orphan crops (Supplementary Table 1).

More complex or polygenic traits, such as yield, are difficult to modulate with gene knockouts. For these traits, a knockout does not always suffice to produce a desired phenotype. Due to the complexity of yield as a trait in crops, thus far very few of the applications of gene editing to orphan crops have directly increased the yield of the crop studied (Supplementary Table 1). To solve this problem, efforts need to be directed to identifying single genes that, when edited, increase yield, employ multiplex editing of multiple genes related to yield, or utilize promoter editing to impact the expression of many genes to achieve the desired phenotypic result. The single gene knockout approach has been utilized in orphan crops, notably to increase the yield of ground cherry (Lemmon et al., 2018). Multiplex approaches to modulating yield may intend to target the CLV/WUS pathway, a highly conserved pathway involved in shoot apical meristem maintenance (Fletcher, 2018; Liu et al., 2021). Multiplexing has been used to edit multiple related genes in *Setaria viridis* (Weiss et al., 2020), though, to date, it has not been used to directly impact yield. Promising recent work in model species suggests that the high editing efficiencies needed to simultaneously target up to 12 genes can be achieved in plants, potentially allowing for the modulation of more complex traits, such as yield, with gene editing (Stuttmann et al., 2021). Additionally, promoter editing of genes related to shoot apical meristem maintenance can be used to modulate yield in plants by modulating the expression levels of multiple related genes (Rodríguez-Leal et al., 2017; Liu et al., 2021).

To upregulate a gene product with CRISPR/Cas, gene editing often requires identifying an endogenous plant pathway, which represses that gene product. Because knockouts and base editing are limited to altering or knocking out the functions of gene products, and knock-ins and prime editing are not yet efficient or common in any crop species, direct upregulation with gene editing is difficult for most non-transgenic approaches. Inhibitors or negative regulators of certain pathways must be identified in order to achieve upregulation with knockout *via* gene editing. A study in *Cucumis sativus*, for example, developed gynoecious plants desired for breeding by knocking out an inhibitor of carpal development (Hu et al., 2017). Non-transgenic upregulation of gene products can also be achieved with promoter editing (Rodríguez-Leal et al., 2017). This, however, requires a well-characterized pathway with multiple regulators, such as the CLE-mediated CLV/WUS pathway (Liu et al., 2021). Currently, however, most plant pathways are not as well described through basic research as CLV/WUS. Alternatively, uORFs may be targeted to upregulate transcription at primary ORFs, though this technique has not yet been widely applied (Zhang et al., 2018). Upregulation of products not expressed naturally *in planta*, such as vitamin A or *Bt* toxin, is currently undesirable with gene editing, as this would require the introduction of an exogenous cistron, leading to the edited orphan crop being regulated as transgenic.

### Unannotated, Polyploid, or Heterozygous Genomes

Genomic resources are a major prerequisite for the efficient application of CRISPR/Cas to orphan crops. However, due to funding constraints and a lack of research interest, and despite the best efforts of researchers, governments, and philanthropic organizations, many orphan crop genomes are currently not sequenced or assembled. Though genome assemblies are available for multiple orphan crops, gene editing has not yet been applied to some major staples, such as tef (Numan et al., 2021) and pigeonpea. If a sequenced genome is unannotated it is difficult, though not impossible, to identify genes of interest with alignment software, such as NCBI BLAST, and exonic regions can be identified utilizing FGNESH+ (Solovyev et al., 2006). Additionally, orphan crop genomes, such as the assembly for African eggplant (*Solanum aethiopicum*), among others published in coordination with the AOCC, are not available on traditional genome browsers [Chang et al., 2018; Song et al., 2019; African Orphan Crops Consortium (AOCC), 2021]. Thus, greater genomic resources, in quality, quantity, and ease of accessibility, are needed before gene editing can be applied to many important orphan staples.

Another constraint in applying CRISPR/Cas to orphan crops is ploidy. Polyploid genomes, such as the economically important wheat or the orphan sweet potato and banana/plantain, are more challenging to sequence and annotate compared to diploid genomes, and present unique challenges in gene editing (Kyriakidou et al., 2018). Because they are difficult to sequence, complex polyploid genomes often lag behind other crops with simpler genomes in the availability and quality of genomic resources (Kyriakidou et al., 2018). A high number of homologs, commonly associated with polyploidy, complicate CRISPR/Cas approaches as multiple gRNAs are required to target all gene copies and transcript variants for a functional knockout. Additionally, when editing multiple homologs, a gene editing system, especially a CRISPR/Cas system with few guides, must have high efficiency to reliably edit all target regions. Despite these challenges, CRISPR has been successfully applied to sweet potato (Wang et al., 2019) and banana/plantain (Kaur et al., 2018; Tripathi et al., 2019; Shao et al., 2020). Alike polyploidy, heterozygosity is also common in orphan crop genomes. Given that there are few orphan crop elite lines or cultivars, there is phenotypic and genetic variation within most species (Cannarozzi et al., 2018; Tadele, 2019). This makes CRISPR/Cas-mediated gene editing more difficult, as grower-preferred varieties and varieties used for research are often genetically dissimilar. Breeding can be utilized to overcome the obstacle of heterozygosity through introgression of alleles from sequenced and annotated lab strains to un-sequenced or unannotated grower-preferred cultivars (Figure 3C).

### Alternative Methods of Crop Improvement

Gene editing is one of many methods of orphan crop improvement. Transgenic approaches overcome the difficulty of upregulation with gene editing and may introduce novel gene products, such as beta carotene, to plants. While effective, transgenic crops are associated with poor public opinion and heavy, costly domestic and international regulation. As a result, there are few accepted transgenic orphan crop accessions in global databases (https://www.isaaa.org/gmapprovaldatabase/). Conventional breeding approaches can also overcome many limitations of gene editing. For example, in conventional breeding, no transformation or regeneration is needed, and there are no constraints associated with mechanistic technicalities, as in transgenic and gene editing approaches. However, the timeline for conventional breeding approaches is significantly longer than that of gene editing. Breeding techniques are also only possible among highly similar organisms (Taagen et al., 2020). Breeding programs are often expensive, and individual breeding events are unpredictable due to an inability to control the passing of undesirable traits to offspring along with desirable ones (Taagen et al., 2020). Given the limitations of gene editing, it is imperative that this technology be utilized alongside existing methods of crop improvement, namely, transgenic and conventional breeding approaches, in order to facilitate the improvement of orphan crops.

## Summary

Orphan crops are distinctly characterized by the lack of funding and scientific research interest associated with them, as evidenced by a limited availability of improved varieties and paucity of basic research (Tadele, 2019). Philanthropic efforts have aided in funding some applied orphan crop research (Gomez et al., 2019), but governments must also recognize the urgency for research associated with orphan crops if significant progress is to be made through gene editing. The process for regulation of a gene edited organism differs among nations, but predominantly, a transgene-free gene edited organism is not constrained by the heavy regulations associated with transgenic organisms (He and Creasey Krainer, 2021). If gene edited organisms are increasingly regulated as transgenic, this regulatory process would serve as a major bottleneck in the development of improved orphan crop varieties through gene editing. Additionally, even if regulations are eased in comparison with transgenic plants, it is imperative to educate the public about the opportunities gene editing with CRISPR/Cas systems provides for agriculture, the environment, and consumers, especially in the developing world. To avoid the societal issues opposing classical transgenic crops, the process, benefits, and limitations of gene editing must be transparent and understood by all.

Gene editing presents an opportunity to circumvent widespread societal distrust of transgenic crops and the heavy regulation and global agricultural inequities associated with it (He and Creasey Krainer, 2021). The rise of the CRISPR/Cas system has allowed for precise, heritable mutations to be made cost-effectively and comes with the benefits of multiplexing, prime editing, and base editing. This system is the first gene editing system to be widely applied in orphan crops. As a result, orphan crop improvement and *de novo* domestication through gene editing are feasible and have been widely demonstrated (Supplementary Table 1). Future mechanistic improvements to CRISPR/Cas systems will increase the capabilities of gene editing in orphan crops. Near PAM-less Cas enzymes and other newly engineered or discovered nucleases will allow unrestricted knockouts and highly targeted base edits *in planta*, further increasing the versatility of gene editing. Current multiplexing strategies only continue to improve in efficiency and scope, especially in model plant species. When further improved and applied in orphan crops, it will be possible to simultaneously edit multiple gene families or target up to 12 genes or more, as was demonstrated in *Arabidopsis thaliana* (Stuttmann et al., 2021). Improvement of the efficiency of HDR and gene knock-ins is imperative, as this currently remains a bottleneck in plants (Huang and Puchta, 2019). These strategies allow mimicking of natural polymorphisms and edit alleles for single amino acid swaps. The improvement of their specificity and efficiency *in planta* is an essential goal.

Methodology in orphan crop gene editing will continue to improve with coordination and research. A greater focus on coordination and collaboration among research efforts should be a primary goal to ensure resources for orphan crops are developed, available, and easily accessible to all. For example, current gRNA design tools are efficient, but many researchers lack the resources and genomic data necessary to work with orphan crops. Basic and applied research cloning systems will inevitably lead to more efficient cloning processes and simpler protocols for designing and building gene editing constructs. In the near future, cloning may even become obsolete due to the decreasing costs and rapid advancements in DNA synthesis technologies. Transformation, though, will continue to be a major bottleneck in gene editing methodology for orphan crops. Crop- specific improvements have been made, but substantially more efficient transformation will be difficult to achieve, based on results from the past decade. Future research must ensure that all crops are able to be transformed easily and that efficient, genotype nonspecific methodologies are developed for orphan crops.

Through better annotation, gene discovery, and further basic study of plant pathways, more gene targets will be identified for future applications of gene editing in orphan crops. With an increased emphasis on funding of orphan crop research and improvement, gene editing will be applied to orphan staples with genome sequences already published. Notably, gene editing has not yet been applied to tef, pigeonpea, mungbean, *Amaranthus* spp. (grain amaranth), African eggplant, hyacinth bean, white fonio, and *Vigna subterranea* (Bambara groundnut), among other orphan staples, despite genomic data being available. Many orphan crops currently do not have genomic data available, but with the declining cost and increasing efficiency of genome sequencing, genomes will be available for many regionally important orphan staples in the near future. The outlook for applications of gene editing to orphan crops is promising, and future research will be greatly expanded in scope by prime editing, promoter editing, knock-ins, and base editing techniques.

The current limitations of gene editing in orphan crops, namely, those associated with the mechanistic constraints of CRISPR/Cas, will be overcome through extensive basic research. As mentioned, transformation and regeneration will likely remain a significant bottleneck for the foreseeable future of orphan crop gene editing (Altpeter et al., 2016). An emphasis must be put on expanding genomic resources and developing improved protocols for orphan crop gene editing and transformation to ensure the rapid improvement of these agronomically important crops through gene editing. It is important to note that gene editing does not exist in a vacuum and that interplay among gene editing and other methods of crop improvement, namely, marker-ssisted breeding, presents an excellent opportunity for expedited crop improvement. Collaboration among basic and applied science and molecular and conventional breeding practices, with input from growers and consumers, is necessary to ensure this end goal.

Current United Nations Population Fund projections see the world population reaching 9.6 billion by 2050, with over 90% of the population growth coming in developing nations, many of which rely primarily on orphan crops as staple foods. In nations projected to see the most population growth, there also exist the greatest rates of hunger and malnutrition today; of the up to 827 million undernourished in 2020, the majority resides in sub-Saharan Africa. These regions are also categorically the most vulnerable to climate change, creating a precarious situation for the future. Improvement of the orphan crops relied upon by the most undernourished regions will abate future strain from population growth and promote resilience to climate change (Tadele, 2014, 2019; Mabhaudhi et al., 2019). This can also lead to more sustainable development by decreasing agricultural land use and input intensity. It is evident that gene editing has the potential to foster this necessary orphan crop improvement, but only if its urgency is recognized through widespread collaboration, ample funding, and the adoption of science-based regulatory processes.

## Author Contributions

MV reviewed the literature, tabulated the data, edited the figures, and wrote the article. KC devised/drafted the article and figures and reviewed/edited the manuscript prior to submission. All authors contributed to the article and approved the submitted version.

## Funding

This review was supported by Grow More Foundation. Grow More Foundation in a 501(c)(3) non-profit NGO with the mission to develop and distribute capacity-building scientific resources to enable plant scientists in developing countries.

## Conflict of Interest

The authors declare that the research was conducted in the absence of any commercial or financial relationships that could be construed as a potential conflict of interest.

## Publisher’s Note

All claims expressed in this article are solely those of the authors and do not necessarily represent those of their affiliated organizations, or those of the publisher, the editors and the reviewers. Any product that may be evaluated in this article, or claim that may be made by its manufacturer, is not guaranteed or endorsed by the publisher.
