# Supplementary material for: Current Advancements and Limitations of Gene Editing in Orphan Crops
Source: Front Plant Sci. 2021 Sep 22;12:742932. doi: 10.3389/fpls.2021.742932 (PMC8493294; doi:10.3389/fpls.2021.742932)
Supplement: Supplementary file 1 [file Table_1.docx]

**­­­Supplementary Table 1.** **Published Applications of Gene Editing in Underutilized and Orphan Crops**

Gene editing has been successfully demonstrated in 22 crops, considered underutilized or orphan crops. To date, a significant portion of published applications are in fruits, mostly *Citrus*. Few applications have been recorded in orphan pulses, pr­­imarily due to low transformation and regeneration efficiencies. Arranged by crop (roots/tubers, cereals, pulses, fruits, and leaf vegetables), target gene(s)/gene edited alleles along with a description of gene editing methodology, are provided with transformation method and outcome. Notably, *PDS* is the most frequently targeted gene in orphan crops. Few gene editing applications to date in orphan crops have utilized promoter editing or base editing, and, currently, there are no published examples of knock-in or prime editing strategies in orphan crops. Most studies in orphan crops have utilized *Agrobacterium­­­*-mediated transformation methodologies, though the starting material for transformation and regeneration is crop-specific. Protoplasts are also widely utilized for their rapid timeframe of isolation and relatively simple transformation procedure. However, there has been limited use of biolistic bombardment. Overall, these studies prove that CRISPR/Cas is a highly versatile editing tool, with great potential in both basic and applied science.

*Phytoene desaturase* (*PDS*); *eukaryotic initiation factor 4E* (*eIF4E*); *Granule-bound starch synthase* (*GBSS*); *Protein targeting to starch 1* (*PTST1*); *Granule-bound starch synthase I* (*GBSSI*); *Starch branching enzyme II* (*SBEII*); *alpha-kafirin encoding k1C* (*k1C*); *Flowering time locus* (*FT*); *Gibberellin 2-oxidase 5* (*GA2ox5*); *Centromere-specific histone 3* (*CENH3*); *MATRILINEAL* (*MTL*); *Domains rearranged methylase 1a* (*Drm1a*); *Domains rearranged methylase 1b* (*Drm1b*); *Male sterility 26* (*Ms26*); *Male sterility 45* (*Ms45*); *4-coumerate ligase* (*4CL*); *Reveille 7* (*RVE7*); *phytosulfokine 1* (*psk1*); *Acetolactate synthase* (*ALS*); *Symbiosis receptor-like kinase* (*SYMRK*); *Non-expressor of pathogenesis-related 3* (*NPR3*); endogenous banana streak virus (eBSV); LCYε (Lycopene epsilon cyclase); *Gibberellin 20-oxidase 2* (*GA20ox2*); *Polyphenol oxidase 4, 5 and 6* (*PPO4, PPO5* and *PPO6*); *Lateral organ boundaries 1* (*LOB1*); *CENTRORADIALIS* (*CEN*); *CENTRORADIALIS 4* (*CEN4*); 9*-cis-EPOXYCAROTENOID DIOXYGENASE4* (*NCED4*); *GDP-L-galactose phosphorylase 1* (*GGP1*); *GDP-L-galactose phosphorylase 2* (*GGP2*); Friable embryogenic callus (FEC); polyethylene glycol (PEG), cassava brown streak virus (CBSV)

* The system mediating gene edits is CRISPR/Cas9 unless otherwise stated.

** “Proof of concept” refers to the first application of gene editing to a particular crop or to any subsequent application that does not modulate an agronomically beneficial trait, instead evaluating the efficacy of a specific methodology of gene editing or transformation.

| **Crop Type** | **Crop** | **Target Gene(s)** | **Gene Edit Description*** | **Transformation Description** | **Outcome of Application**** | **Citation** |
| --- | --- | --- | --- | --- | --- | --- |
| Roots/Tubers | *Manihot esculenta* (cassava) | *PDS* | Functional knockout | *Agrobacterium*-mediated shoot explant (FEC) co-cultivation | Proof of concept (first application)/albino phenotype | (Odipio et al., 2017) |
|  |  | two *eIF4E* isoforms |  |  | Resistance to potyviruses (CBSV) | (Gomez et al., 2019) |
|  |  | *GBSS* and *PTST1* |  |  | More digestible starch | (Bull et al., 2018) |
|  | *Ipomoea batatas* (sweet potato) | *GBSSI* and *SBEII* | Functional knockout | *Agrobacterium*-mediated shoot explant co-cultivation | Proof of concept (first application)/more digestible starch | (Wang et al., 2019) |
|  | *Dioscorea* spp. (yam) | *PDS* | Functional knockout | *Agrobacterium*-mediated nodal explant co-cultivation | Proof of concept (first application)/albino phenotype | (Syombua et al., 2021) |
| Cereals | *Sorghum bicolor* (sorghum) | *k1C* gene family | Functional knockout of gene family without multiplexing | *Agrobacterium*-mediated immature embryo co-cultivation | More digestible protein | (Li et al., 2018a) |
|  |  | *FT* and *GA2ox5* | Functional knockout |  | Proof of concept for heritable gene edits/delayed flowering time | (Char et al., 2020) |
|  |  | *CENH3* | Functional knockout |  | Proof of concept for improved transformation | (Che et al., 2018) |
|  |  | *PDS* | Functional knockout | Biolistic bombardment of immature embryos | Proof of concept for transformation method/albino phenotype | (Liu et al., 2019a) |
|  | *Oryza spp.* (African rice) | multiple domestication-related genes | Functional knockout | *Agrobacterium*-mediated immature embryo co-cultivation | Improvement of domestication related traits including yield and plant architecture | (Lacchini et al., 2020) |
|  | *Oryza alta* (allotetraploid wild rice) |  |  |  |  | (Yu et al., 2021) |
|  | *Setaria italica* (foxtail millet) | *PDS* | Functional knockout | PEG-mediated protoplast | Proof of concept (first application) | (Lin et al., 2018) |
|  |  | *MTL* | Functional knockout | *Agrobacterium*-mediated immature embryo co-cultivation | Generation of haploid inducer line | (Cheng et al., 2021) |
|  | *Setaria viridis* (green foxtail) | *Drm1a* and *Drm1b*; *Ms26* and *Ms45* | Functional knockout mediated by CRISPR/Cas9_Trex2 | *Agrobacterium*-mediated immature embryo co-cultivation | Proof of concept for multiplexing with Cas9_Trex2 in plants | (Weiss et al., 2020) |
| Pulses | *Cicer arietinum* (chickpea) | *4CL* and *RVE7* | Functional knockout | PEG-mediated protoplast | Proof of concept (first application) | (Badhan et al., 2021) |
|  | *Vigna unguiculata* (cowpea) | *SYMRK* | Functional knockout | *Agrobacterium*-mediated hairy root infection | Proof of concept (first application)/altered root morphology | (Ji et al., 2019) |
| Fruits | *Citrullus lanatus* (watermelon) | *psk1* | Functional knockout | *Agrobacterium*-mediated cotyledon explant co-cultivation | Resistance to fungal infection | (Zhang et al., 2020) |
|  |  | *ALS* | Base editing mediated by a Cas9-cytidine deaminase fusion protein |  | Herbicide tolerance | (Tian et al., 2018) |
|  |  | *PDS* | Functional knockout |  | Proof of concept (first application)/albino phenotype | (Tian et al., 2017) |
|  | *Cucumis sativus* (cucumber) | *eIF4E* | Functional knockout | *Agrobacterium*-mediated cotyledon explant co-cultivation | Proof of concept (first application)/ resistance to viruses | (Chandrasekaran et al., 2016) |
|  |  | *WIP1* |  |  | Gynoecious plants | (Hu et al., 2017) |
|  | *Cucumis melo* (melon) | *PDS* | Functional knockout | *Agrobacterium*-mediated cotyledon explant co-cultivation | Proof of concept (first application)/albino phenotype | (Hooghvorst et al., 2019) |
|  | *Musa* spp. (banana and plantain) | *PDS* | Functional knockout | *Agrobacterium*-mediated embryogenic cell suspension co-cultivation | Proof of concept (first application)/albino phenotype | (Kaur et al., 2018) |
|  |  | eBSV |  |  | Inactivation of eBSV | (Tripathi et al., 2019) |
|  |  | *GA20ox2* |  |  | Semi-dwarf plants | (Shao et al., 2020) |
|  |  | LCYε |  |  | β-carotene-enriched fruits | (Kaur et al., 2020) |
|  | *Theobroma cacao* (cacao) | *NPR3* | Functional knockout | *Agrobacterium*-mediated transient leaf explant infiltration | Proof of concept (first application)/resistance to fungal infection | (Fister et al., 2018) |
|  | *Solanum melongena* (eggplant) | *PPO4*, *PPO5,* and *PPO6* | Functional knockout | *Agrobacterium*-mediated cotyledon explant co-cultivation | Proof of concept (first application)/decreased flesh browning | (Maioli et al., 2020) |
|  | *Citrus* (citrus) | *LOB1* | Functional knockout | *Agrobacterium*-mediated epicotyl explant cocultivation | Resistance to bacterial infection (citrus canker) | (Jia et al., 2017) |
|  |  | *LOB1* promoter | Promoter editing |  |  | (Peng et al., 2017) |
|  |  | Pth4A element of the *LOB1* promoter | Promoter editing |  |  | (Jia et al., 2016) |
|  |  | *LOB1* | Functional knockout mediated by CRISPR/Cpf1(Cas12a) |  |  | (Jia et al., 2019) |
|  |  | *PDS* | Functional knockout | *Agrobacterium*-mediated transient leaf explant infiltration | Proof of concept (first application)/albino phenotype | (Jia et al., 2014) |
|  |  |  |  | *Agrobacterium*-mediated epicotyl explant co-cultivation | Proof of concept for novel promoter/albino phenotype | (Zhang et al., 2017) |
|  |  |  |  | *Agrobacterium*-mediated embryogenic cell culture co-cultivation | Proof of concept for transformation method/albino phenotype | (Dutt et al., 2020) |
|  |  |  |  | PEG-mediated protoplast | Proof of concept (first application in protoplasts) | (Huang et al., 2020) |
|  | *Fortunella hindsii* (Hongkong kumquat) | *PDS* | Functional knockout | *Agrobacterium*-mediated epicotyl explant co-cultivation | Proof of concept (first application)/albino phenotype | (Zhu et al., 2019) |
|  | *Physalis pruinosa* (groundcherry) | multiple domestication-related genes | Functional knockout | *Agrobacterium*-mediated hypocotyl explant co-cultivation | Improvement of domestication related traits including yield, growth habit and morphology | (Lemmon et al., 2018) |
|  | *Solanum pimpinellifolium* (wild tomato) |  |  | *Agrobacterium*-mediated leaf explant co-cultivation |  | (Zsögön et al., 2018) |
|  | *Actinidia chinensis* (kiwifruit) | *PDS* | Functional knockout | *Agrobacterium*-mediated leaf explant co-cultivation | Proof of concept (first application)/bleached phenotype | (Wang et al., 2018) |
|  |  | *CEN* and *CEN4* | Functional knockout |  | Rapid flowering time and more compact plants | (Varkonyi‐Gasic et al., 2019) |
| Leaf Vegetables | *Lactuca sativa* (lettuce) | *NCED4* | Functional knockout | *Agrobacterium*-mediated cotyledon explant co-cultivation | Proof of concept (first application)/high temperature germination | (Bertier et al., 2018) |
|  |  | uORFs of the *GGP1* and *GGP2* | uORF editing |  | Increased vitamin C content | (Zhang et al., 2018) |
